# Effects of Therapeutic Hypothermia on Normal and Ischemic Heart

**DOI:** 10.3389/fcvm.2021.642843

**Published:** 2021-02-15

**Authors:** Kelly P. Yamada, Taro Kariya, Tadao Aikawa, Kiyotake Ishikawa

**Affiliations:** Cardiovascular Research Institute, Icahn School of Medicine at Mount Sinai, New York, NY, United States

**Keywords:** hypothermia, myocardial infarction, infarct size, endovascular, physiology, cardiac function, clinical trial, preclinical

## Abstract

Therapeutic hypothermia has been used for treating brain injury after out-of-hospital cardiac arrest. Its potential benefit on minimizing myocardial ischemic injury has been explored, but clinical evidence has yet to confirm positive results in preclinical studies. Importantly, therapeutic hypothermia for myocardial infarction is unique in that it can be initiated prior to reperfusion, in contrast to its application for brain injury in resuscitated cardiac arrest patients. Recent advance in cooling technology allows more rapid cooling of the heart than ever and new clinical trials are designed to examine the efficacy of rapid therapeutic hypothermia for myocardial infarction. In this review, we summarize current knowledge regarding the effect of hypothermia on normal and ischemic hearts and discuss issues to be solved in order to realize its clinical application for treating acute myocardial infarction.

## Introduction

The impact of temperature on human biology has been researched extensively and a number of experimental studies have shown that lowering body temperature is capable of protecting tissues from injury (1–7). To take advantage of this protective effect, the concept of therapeutic hypothermia (TH) has been developed and tested in patients with various diseases. Different levels of hypothermia including mild (32–35°C), moderate (28–32°C), severe (20–28°C), and profound (<20°C) were proposed (8). Clinical application of TH for acute diseases is mainly limited to mild and moderate hypothermia due to technical challenges and increased risks of arrhythmias at lower temperature range. Meanwhile, deeper TH has been applied to protect organs during circulatory arrest for cardiac and aortic surgeries (9). Successful demonstration of injury limitation in experimental studies and early clinical trials (10, 11) prompted researchers to use TH also for ST-elevation myocardial infarction (STEMI). However, much of the current clinical evidence of TH in organ protection was derived from studies that focused on neurological injury, while those focusing on the ischemic heart remain limited. Whereas reperfusion has already taken place in the brains of patients after resuscitation for out-of-hospital cardiac arrest (OHCA), the circumstances of STEMI are unique in that hypothermia can be applied prior to the reperfusion of ischemic myocardium. It therefore offers targeting of reperfusion injury in addition to post-reperfusion injury. The emergence of new devices and techniques that allow rapid cooling of the heart opens the door for discussion on whether priority should be placed on the attainment of a target temperature or on more rapid reperfusion. In this review, we summarize our current knowledge related to the impact of TH on the heart and discuss its potential benefit for treating STEMI.

## ECG Changes and Arrhythmias Associated With TH

A decrease in heart rate is the most consistently reported electrophysiological change associated with TH in both normal and ischemic hearts (12–15). In addition to a reduced sinus rate, atrial and ventricular conduction velocities seem to decrease under hypothermia as represented by prolongation of PR, QRS, and QT intervals (16–18). Whether profound bradycardia during TH for MI would require intervention remains unclear, but bradycardia was apparently a favorable marker for patients undergoing TH after resuscitation following OHCA (14). Although it is unknown if lower heart rate in this study actually contributed to the good outcome or it was just reflecting less myocardial injury, bradycardia may be treated conservatively unless there is an evidence of organ hypo-perfusion.

The J wave is a characteristic ECG change found in some hypothermic patients. Up to 30% of patients after OHCA presented with J waves during TH and its prevalence was found to be higher in patients with STEMI (19). In cases of accidental hypothermia, the J wave was observed more frequently in patients having lower body temperatures (20, 21), suggesting a temperature dependent increase in its appearance. Interestingly, there was also an inverse correlation between temperature and the size of the J wave (21). These ECG changes sometimes mimic those of STEMI. In fact, Rolfast et al. (19) reported ST changes during TH in some OHCA patients who lacked actual coronary occlusion, which was confirmed by coronary angiograms.

Potential increase in incidence of atrial and ventricular arrhythmias has been a concern for applying TH in STEMI patients. In the COOL AMI EU pilot trial, which used an endovascular cooling method, the incidence of atrial fibrillation was more common in the TH group (32%) compared to the control group (8%, *P* = 0.07) (22). Using naïve pigs, Manninger et al. (18) found that TH prolongs the effective atrial refractory period at 33°C, which was accompanied by an increase in pacing-induced atrial fibrillation. However, the serum potassium level was decreased during hypothermia in this study, suggesting a potential influence of the dysregulated electrolyte. In contrast, in the *post hoc* analysis of a Targeted Temperature Management (TTM) trial, TH was not associated with the incidence of atrial fibrillation in patients with new-onset STEMI (23). Combined analysis of the RAPID MI-ICE and the CHILL-MI trials also exhibited no difference in atrial fibrillation incidence (24). Within the temperature range used in STEMI cooling studies, no significant increase in ventricular arrhythmias has been reported (22, 24), a finding consistent with animal studies (25). Taken together, mild TH seems not to significantly increase the occurrence of arrhythmia in general STEMI patients, but there could be a subpopulation of patients more prone to the development of arrhythmias, such as those with electrolyte dysregulation. Because hypothermia can dysregulate electrolyte balance through volume shift and by influencing kidney excretion (26), careful electrolyte monitoring is likely important.

## Impact of TH on Cardiac Function

In non-diseased hearts, several *ex vivo* studies reproducibly showed that hypothermia increased cardiac contractility (27–30). Despite increasing contractility, however, myocardial oxygen consumption remained similar and hypothermia was thus believed to improve myocardial energy efficiency (27, 31). The contractility increase was accompanied by prolonged systolic time (27, 32), resulting in a delay in achieving end-systole during ventricular ejection. Because of the prolonged systole, systolic functional parameters that include time component (e.g., maximum dP/dt, tissue Doppler velocity) did not necessary indicate improvement, whereas time-independent contractility parameters such as Emax or slope of end-systolic pressure-volume relationship generally showed an increase (12, 27–30). Some conflicting results exist for *in vivo* studies showing decreases in stroke volume or cardiac output (33, 34), but this was likely associated with complex biological interactions such as neuromodulation (35) and altered vascular resistance (36, 37).

As discussed above, heart rate slows by TH and helps to compensate for reduced diastolic time associated with prolonged systole. But even with lower heart rate, diastolic functional parameters are usually impaired under hypothermic conditions (37). Both active relaxation, as assessed by cardiac relaxation time constant, tau, or minimum dP/dt, and left ventricular stiffness, as assessed by left ventricular end-diastolic pressure or end-diastolic pressure-volume relationships, can be impaired by cooling (12, 38, 39). Myocardial stiffening associated with lower myocardial temperature might be responsible for increase in end-systolic and end-diastolic elastance. Meanwhile, increasing the heart rate by pacing has been shown to impair systolic function and also to worsen diastolic dysfunction (40). These results suggest mechanistic importance of prolonged systole for maintaining systolic function.

There are also limited data on the impact of hypothermia on cardiac function during myocardial ischemia, but available data suggest that functional changes in response to hypothermia are generally similar to those of the normal heart (41, 42). Interestingly, some previous studies before the reperfusion era showed that myocardial function (cardiac output and left ventricular stroke work) was better in hypothermia-treated animals after rewarming compared to the normothermic animals, despite the absence of reperfusion (33, 43). Whether hypothermia and rewarming also improves cardiac function without coronary reperfusion as these authors suggested or it was associated with rewarming induced vasodilation remains unclear. In either case, more data on rewarming after reperfusion is necessary to devise appropriate exit strategies for cardiac TH.

In summary, TH seems to have positive or at least neutral effects on contractility, but negative effects on diastolic function. Yet, studies that examined the impact of TH on *in vivo* heart function are limited and data are not always consistent with *ex vivo* findings. These are likely related to the difference in experimental settings including method and speed of cooling, animal species, anesthesia, and means of functional assessment.

## Mechanisms of Infarct Reduction

A large body of data on protective mechanisms associated with hypothermia derives from studies in neurons or in the arrested human heart at much lower temperatures. Limited studies have investigated mild hypothermia mediated protection in the myocardial ischemia setting (44–50). Nevertheless, available studies report similar mechanisms in the ischemic myocardium to those found in neuron studies in preventing post-reperfusion injury (8). However, TH can be applied prior to reperfusion in STEMI and it may offer an additive benefit by alleviating ischemia before reperfusion and also by reducing reperfusion injury at the early phase of reperfusion. Potential mechanisms of infarct size reduction by TH are discussed here.

### Alleviating Ischemia

As discussed above, cardiac contractility is expected to be preserved during mild TH whereas myocardial energy efficiency is improved. TH also reduces heart rate. These are expected to reduce myocardial oxygen consumption related to the pump function (mechanical work) and alleviate ischemia progression. TH can also affect cardiac metabolism. Whole body metabolism and oxygen consumption decrease substantially as the temperature decreases (51). The relationship between temperature and oxygen consumption is likely non-linear, with a greater reduction of oxygen consumption in the first few degrees from normothermia (52, 53). It is expected that the ischemic myocardium also follows this relationship, and thus oxygen demand as well as tissue metabolism are likely suppressed early after TH induction. In a rabbit heart, initiation of epicardial cooling before MI preserved tissue adenosine triphosphate (ATP) and glycogen in the ischemic myocardium 20 min after MI (44). Reduced metabolism would also alleviate cellular acidosis, which can trigger cell death (54). However, it remains uncertain if and to what extent reductions in metabolism and oxygen consumption would offer benefit in the already ischemic myocardium, since energy stores are likely depleted by the time TH is initiated, unless started immediately after the onset of ischemia. Using a dog isolated heart, Jones et al. (55) reported that despite a 50% reduction in both ATP utilization and anaerobic glycolytic ATP production, energy deprivation could not be prevented and all hearts resulted in contracture-rigor, although with some delay in the hypothermia treated hearts. Thus, it is convincing that mechanisms other than mere reduction in energy consumption play important roles in myocardial protection during ischemia and reperfusion processes. That being said, slowing of energy utilization might offer large benefits to patients who have rich collateral supply to the ischemic myocardium, those with partially recanalized coronary, or those who arrived hospital early after the ischemia onset.

### Reducing Reperfusion Injury

Reperfusion injury is estimated to cause around 50% of total myocardial injury in MI (56). Various mechanisms contribute to reperfusion injury and hypothermia seems to inhibit many of these pathological processes at the cellular level (26, 57). For example, TH has been shown to reduce cellular calcium load after reperfusion (58), which causes cell necrosis via the opening of mitochondrial permeability transition pores. Hypothermia-mediated apoptosis inhibition has been shown in many studies of neuron injury (59, 60), but data in myocardial ischemia-reperfusion injury remain scarce. *In vitro* studies using cardiomyocyte cell lines indicate that apoptosis of these cells at reperfusion following oxygen/energy deprivation is suppressed by TH (61, 62). Using an isolated rabbit heart, Ning et al. (63) reported reduced apoptosis in hearts maintained at 30°C during ischemia compared to those maintained at 34°C, but unfortunately, this study did not include settings at higher temperatures. Upon ischemia-reperfusion, rapid increases in oxygen radicals induce tissue oxidative stress, which has been shown to be inhibited by TH in both the heart (64) and neurons (65). Additionally, maintenance of cellular membrane integrity by hypothermia might prevent cellular edema. This is supported by a study that reported reduced myocardial edema after TH which was detected by magnetic resonance imaging in a pig model of ischemia-reperfusion (66). Hypothermia has also been reported to suppress post-ischemic inflammation via reduction of pro-inflammatory cytokine release (67) and local immune cell activation (66).

At the molecular level, Yang et al. (68) showed that hypothermia (35°C) increases extracellular signal-regulated kinase (ERK) activity in isolated rabbit hearts, the inhibition of which abolished the beneficial effects on infarct size. Using rat isolated hearts, Mochizuki et al. (46) reported that nitric oxide (NO) and phosphatidylinositol 3'-kinase (PI3K) are the key molecules in hypothermia (34°C) mediated infarct size reduction. Other studies also reported that increased AKT phosphorylation (47), reduced p53 expression, and increased heme-oxygenase 1 (50) play major roles in hypothermia-mediated reduction of reperfusion injury.

**Figure 1:**
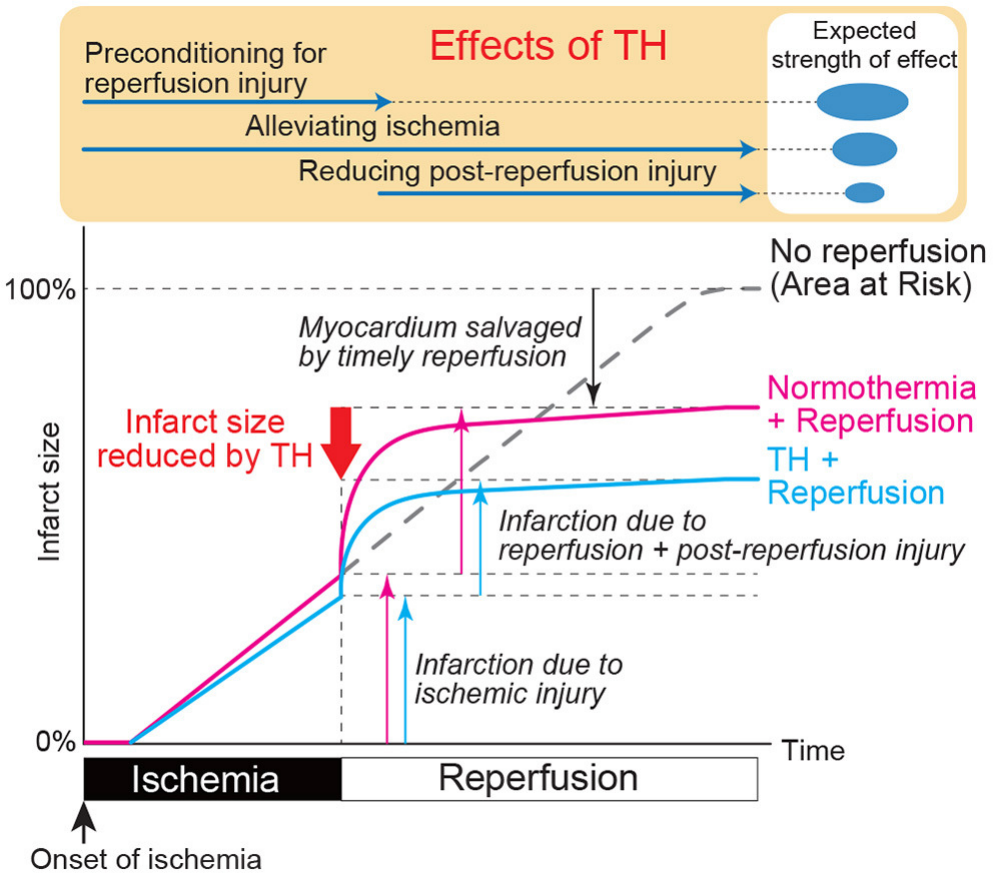
Expected impact of therapeutic hypothermia on myocardial ischemia and reperfusion injury. The pink line shows the time course of myocardial injury associated with ischemia and reperfusion. X axis indicates time after ischemia onset and Y axis indicates infarcted myocardium relative to ischemia area-at-risk. Largest injury is expected just after the reperfusion. Blue line shows the expected time course of myocardial injury with TH when applied at the same time as the ischemia. Depending on when TH is started, the line may diverge at that time point. Preclinical data suggest that TH started at the time of reperfusion does not reduce myocardial injury. Therefore, TH during ischemia likely has preconditioning effect that prepares the myocardium for reperfusion. Expected strength of this preconditioning effect, ischemia alleviation, and attenuation of reperfusion injury are shown in the top of the Figure.

Interestingly, several reports indicate that hypothermia initiated after reperfusion does not reduce infarct size, whereas its initiation before reperfusion does so even if it is delayed from onset of the ischemia (69, 70). These results suggest that hypothermia may precondition the heart to alleviate injury associated with the very acute phase of reperfusion, which is expected to be the major portion of total reperfusion injury as shown in Figure 1. This might be the unique feature of hypothermia that allows alteration of ischemic myocardial wall property i.e., temperature, through endocardial transmission in the absence of coronary recanalization, which is not possible by pharmacological approaches. It remains uncertain, however, what mechanisms underlie in this protective preconditioning effect, since most of the previous reports studied myocardial molecular changes after the reperfusion.

## Impact of TH On Coronary Flow – Microvascular Obstruction

In addition to the reduction of infarct size, TH has been shown to offer beneficial effects on post-reperfusion coronary flow. In a series of experiments, Hale et al. (71, 72) reported that direct ice bag cooling of the rabbit heart initiated at the peri-reperfusion period reduced the no-reflow phenomenon without a change in acute infarct size. This result is supported by a study in pigs by Gotberg et al. (73), which demonstrated significant reduction of microvascular obstruction in the ischemic area as assessed by single photon emission computed tomography. However, clinical trials have yet to confirm these findings in humans and one randomized trial failed to find differences in the size of microvascular obstruction areas between patients treated with hypothermia and controls (74). More recently, Testori et al. (75) also reported that microvascular obstruction area assessed by cardiac magnetic resonance imaging was not different between the patients treated with and without TH 4 days after the onset of STEMI.

## TH For Cardiogenic Shock Associated With STEMI

As discussed above, mild TH increases cardiac contractility. Vasoconstriction of the peripheral vasculature increases systemic vascular resistance (36, 37) and raises arterial pressure. In addition, systemic hypothermia reduces metabolic demand of the whole body (76), which improves supply/demand of the non-cardiac organs. Therefore, theoretically, hypothermia would be an appropriate therapy for cardiogenic shock. Clinical studies in cardiogenic shock patients after cardiac surgery reported an increase in venous oxygen saturation upon hypothermia, indicating improved whole body oxygen supply/demand (12, 77, 78). Meanwhile, *post-hoc* analysis of TTM trial found that patients that required high dose of vasopressors were more common in the 33°C group than 36°C group (79). Whether this would be the same for patients in cardiogenic shock remains unclear. In a recent randomized trial involving 40 patients with cardiogenic shock, hypothermia failed to improve cardiac power as well as clinical outcome (80). Because only around half of the patients were STEMI-associated cardiogenic shock, more studies are needed to determine if hypothermia is safe and efficacious in treating STEMI-associated cardiogenic shock.

## Animal Studies of TH For Reducing Myocardial Infarct Size

Infarct size reduction by TH has been studied in various animal species with variety of cooling methods, ischemia duration, cooling duration, and timing of cooling initiation. While small animal studies are useful in studying the mechanisms, large differences in body size, morphology, and heart size that allow much faster cooling may not fully represent cooling conditions in a clinical setting. Large animal models offer a simulation of hypothermia in clinically relevant conditions and also allow endovascular or intracoronary cooling approaches, which are not feasible in small animals. A summary of representative preclinical experiments in large animals that examined the impact of hypothermia on infarct size is provided in Supplementary Table 1 (33, 42, 64, 66, 69–73, 81–97). In general, studies that initiated cooling prior to reperfusion have shown reduction of infarct size, whereas studies that initiated cooling just prior or after reperfusion tended to show no benefit, regardless of the cooling method. The majority of these studies only looked at the acute impact of TH and there are very few data on the impact of rewarming on infarct size. Accordingly, whether acute benefits on the infarct size can be maintained throughout the chronic phase remain unclear.

## Clinical Trials of TH For STEMI

Similar to preclinical studies, various approaches and devices have been employed for the controlled and efficient cooling of patients in clinical trials targeting STEMI (Table 1) (22, 74, 75, 98–109). The ideal cooling method for STEMI application would be one that offers rapid cooling with the ability to control body temperature throughout the temperature management period, from initiation through the rewarming phase. The ideal method would also be minimally invasive and implemented easily, in an ambulance if necessary, and without significant side effects. Shivering in response to cooling in awake patients is another factor that needs attention because it can significantly increase body oxygen demand and slow cooling speed. Counter-heating of the skin during TH seems to be effective in reducing the shivering (110, 111), which is obviously not available for surface cooling methods and necessitate anti-shivering drug administrations. There is currently no single method that meets all above ideal features, leaving each with its own advantages and disadvantages (Table 2 and Supplementary Table 2).

**Table 1:** Summary list of clinical therapeutic hypothermia studies targeting myocardial infarction.

**Author**	**References**	**Hypothermia method**	**Patient no**.	**Target temp (^**°**^C)**	**TH infarct size (%LV)**	**Control infarct size (%LV)**	**Significant**
Dixon et al.	(93)	Endovascular cooling	42	33	2%	8%	No
O'Neill et al.	(101)	Endovascular cooling	392	33	14.1%	13.8%	No
Kandzari et al.	(97)	Endovascular cooling	18	33.5	4.0% (Day 30)	No control	NA
Ly et al.	(96)	Surface (Arctic Sun)	11	32–34	23%	No control	NA
Knafelj et al.	(105)	Surface + IV saline	72	32–34	NA	NA	NA
Wolfrum et al.	(103)	Surface + IV saline	33	32–34	NA	NA	NA
Schefold et al.	(104)	Surface + IV saline	62	33	NA	NA	NA
Koreny et al.	(100)	Surface (TheraKool)	111	32–34	NA	NA	NA
Götberg et al.	(95)	Endovascular cooling	20	33	13.7%	20.5%	No
Testori et al.	(99)	Surface + endovascular + IV saline	19	35	NA	No control	NA
Erlinge et al.	(102)	Endovascular + IV saline	120	33	40.5%	46.6%	No
Nichol et al.	(98)	Peritonial cooling	54	32.5	16.1%	17.2%	No
Otterspoor et al.	(94)	Intracoronary	10	−6°C Body temp	NA	No control	NA
Noc et al.	(16)	Endovascular cooling	50	32	16.7%	23.8%	No
Testori et al.	(75)	Surface + endovascular + IV saline	101	34	22% (Day4)	22% (Day4)	No

*More detailed information of the studies are provided in Supplementary Table 2. CMR, cardiac magnetic resonance; IV, intravenous; LAD, left anterior descending artery; LV, left ventricle; MACE, major advanced cardiac events; MI, myocardial infarction; NA, not available; OHCA, out of hospital cardiac arrest; SPECT, single-photon emission computerized tomography; STEMI, ST elevation myocardial infarction; TH, therapeutic hypothermia*.

**Table 2:** Approaches to cool down the heart.

	**Speed**	**Access**	**Earliest starting**	**Temperature control**	**Technical feasibility**	**Other**
**Systemic**						
IV cold fluid	++	Periferal vein	Ambulance	Low	High	Potential lung congestion
						Evidence suggests negative impact on clinical outcome
Surface cooling	+	Percutaneous	Ambulance	Low	High	Speed depends on the cooling device
Endovascular	+++	Central vein	ER	High	Medium	
Peritoneal	+++	Peritoneal	ER	Medium	Low	Increased adverse events
Inhalation	++	Intubation	ER	Low	Medium	Limited experience
ECMO	++++	Central artery and vein	ER	High	Low	Increased risk of bleeding
**Local**						
Intracoronary	++++	Artery	Cathlab	Low	Low	Myocardial temperature may be different from coronary temperature
Direct cooling	++++	Open chest	Surgical room	Low	Low	

*ECMO, extracorporeal membrane oxygenation; ER, emergency room; IV, intravenous*.

Ly et al. (101) used surface cooling and reported that target temperature of 34.5°C could be achieved in an average of 79 min. Although the trial did not find safety issues, no follow-up studies have been conducted, likely due to advances in cooling technology that offered much faster cooling. Peritoneal cooling was tested in 54 STEMI patients who were randomized to hypothermia (*n* = 28) and control (*n* = 26) (103). The study demonstrated that peritoneal cooling offers rapid cool-down of patients. However, there was no reduction of infarct size, whereas some concerns regarding increased safety issues were noted. Intracoronary hypothermia is another invasive approach that has been shown to be effective in rapidly and locally lowering the myocardial temperature (112). In a recent trial conducted in Europe, 10 patients were treated with intracoronary hypothermia by injecting room temperature saline through the coronary balloon catheter wire lumen, which was followed by 4°C saline injection after reperfusion (99). The authors reported arrhythmic events in patients with inferior MI, but not in the anterior MI, concluding that it was safe and feasible in patients with anterior MI. Follow up randomized studies are currently recruiting patients in Europe with an expected enrollment of 200 patients [Clinicaltrials.gov identifier NCT03447834 (113) accessed on Jan 15th, 2021]. Endovascular cooling has been the most popular method in the past STEMI trials likely owing to its relatively fast cooling, feasible application, and without requiring substantial amount of fluid loading. Some of these trials examined feasibility and safety (100, 102, 104), which were successfully confirmed, but none of the subsequent efficacy trials were able to meet the primary efficacy endpoints (22, 74, 75, 98, 106). More recently, Dae et al. (90) combined the data of 6 previous randomized clinical trials that used endovascular cooling method and analyzed the infarct size at 1 month after MI on a patient basis. Overall, 629 patients were included in the analysis and the study identified that anterior MI patients who were cooled to below 35°C at the time or reperfusion did show reduced infarct size over the control group, whereas other patient populations failed to show infarct size reduction. These results strongly suggest that rapid cooling to below 35°C is necessary, and patients with larger MI benefit most from TH. As we illustrated in Figure 1, we expect that the inhibition of reperfusion injury offers major benefit in TH. If 35°C is the threshold temperature to inhibit reperfusion injury, some patients may require additional time to reach this point before reperfusion. Existing data is insufficient to determine if delaying reperfusion for a short period to achieve target temperature below 35°C offers more benefit than immediate reperfusion. Currently, a clinical trial that aims to test the safety of new powerful endovascular cooling device is planned in the US [Clinicaltrials.gov identifier NCT03361995 (114) accessed on Jan 15th, 2021] and might provide more information on the temperature threshold at the time of reperfusion.

## Potential Reasons for Lack of Efficacy In Clinical Trials

Although blood temperature is expected to correlate with cardiac temperature, direct monitoring of ischemic myocardial temperature in STEMI setting is challenging. Moreover, cooling speed of the heart and other organs varies depending on the employed method (115). Therefore, inconclusive results in above discussed studies might be associated with insufficient lowering of the ischemic myocardium in contrast to the reported measured temperature. Unlike animal experiments, infarct size measurement in humans relies on imaging modalities and these indirect measures of infarct size assessment could be the source of measurement errors that can obscure the results (75). Additionally, some of the patients presenting STEMI might already have reperfusion at the time of the first coronary angiogram (116). Based on preclinical studies and the recent report by Dae et al. (90), these patients may not benefit from TH since reperfusion has already taken place. The efficacy of some drugs is known to be impaired at low temperature and these drug interactions need careful attention (26). There is a possibility that TH might have synergistic effects when combined with other therapies directed at reducing myocardial infarction (117), and this area remains largely unexplored yet.

## Conclusions and Future Perspectives

Available evidence suggests that TH has the potential to reduce myocardial ischemic injury in humans. However, randomized clinical trials have yet to prove promising results in preclinical studies. Compared to the large number of studies focusing on TH for post-resuscitation brain injury or myocardial protection for surgery, that of alleviating myocardial reperfusion injury remains much less. Accordingly, there remain many questions that are only vaguely answered. These include: (1) Optimal target temperature for STEMI application; (2) Optimal TH method; (3) Whether target temperature needs to be achieved prior to reperfusion; (4) Optimal duration of hypothermia; (5) Mechanisms of myocardial protection; (6) Optimal target patient population; and (7) Optimal protocol for rewarming. Emergence of new devices that allow faster cooling may help to better define some of these questions and lead to positive results in forthcoming clinical trials.

## Author Contributions

KI drafted the manuscript. All other authors critically edited the manuscript and provided key information on this review article. All authors contributed to the article and approved the submitted version.

## Conflict of Interest

The authors declare that the research was conducted in the absence of any commercial or financial relationships that could be construed as a potential conflict of interest.
